# Single Cylinder-Type Piezoelectric Actuator with Two Active Kinematic Pairs

**DOI:** 10.3390/mi9110597

**Published:** 2018-11-15

**Authors:** Ramutis Bansevicius, Jurate Janutenaite-Bogdaniene, Vytautas Jurenas, Genadijus Kulvietis, Dalius Mazeika, Asta Drukteiniene

**Affiliations:** 1 Institute of Mechatronics, Kaunas University of Technology, K. Donelaicio st. 73, LT-44249 Kaunas, Lithuania; ramutis.bansevicius@ktu.lt (R.B.); vytautas.jurenas@ktu.lt (V.J.); 2 Department of Information Systems, Vilnius Gediminas Technical University, Sauletekio al. 11, LT-10223 Vilnius, Lithuania; genadijus.kulvietis@vgtu.lt (G.K.); dalius.mazeika@vgtu.lt (D.M.); asta.drukteiniene@gmail.com (A.D.)

**Keywords:** piezoelectric actuators, positioning, trajectory control, numerical analysis, trajectory planning

## Abstract

There is an ever-increasing demand for small-size, low-cost, and high-precision positioning systems. Therefore, investigation in this field is performed to search for various solutions that can meet technical requirements of precise multi-degree-of-freedom (DOF) positioning systems. This paper presents a new design of a piezoelectric cylindrical actuator with two active kinematic pairs. This means that a single actuator is used to create vibrations that are transformed into the rotation of the sphere located on the top of the cylinder and at the same time ensure movement of the piezoelectric cylinder on the plane. Numerical and experimental investigations of the piezoelectric cylinder have been performed. A mathematical model of contacting force control was developed to solve the problem of positioning of the rotor when it needs to be rotated or moved according to a specific motion trajectory. The numerical simulation included harmonic response analysis of the actuator to analyze the trajectories of the contact points motion. A prototype actuator has been manufactured and tested. Obtained results confirmed that such a device is suitable for both positioning and movement of the actuator in the plane.

## 1. Introduction

Over recent decades, there has been an ever-increasing demand for the positioning systems with several degrees-of-freedom that would be faster, more accurate, and reliable, yet more compact at the same time. Piezoelectric devices are preferable when solving this type of problems because of the high precision, quick response, and low cost. Such advantages of piezoelectric devices are applicable in different fields, where nanometric scale resolution is required as, for example, in microscopes, laser systems, precision positioning systems [1,2,3,4], etc.

Precise positioning is a complicated problem because it affects the accuracy and proper functioning of the mechanism. Various precision positioning systems are proposed that employ piezoelectric actuators and flexible hinges to move or rotate the platform and to achieve the desired motion [1,2]. However, in most cases piezoelectric actuators are used as single degree-of-freedom (DOF) devices despite that there is a high demand for multiple DOF systems. Multiple DOF piezoelectric systems usually require one actuator for each degree-of-freedom, where each actuator requires its own piezoelectric transducer to transform the electrical input signal into a mechanical output. It is difficult to achieve high-resolution systems applying such design principle.

Cai et al. proposed a 6-DOF system, which is a combination of two 3-DOF precision positioning stages [1]. The first stage operates on the plane and ensures translation in *X* and *Y* directions and rotation about *Z* axis. The second stage is out-of-plane and has the first stage mounted on it. Both stages use piezoelectric actuators in combination with flexure hinges to achieve high accuracy. However, the structure of the proposed system is quite complicated. Lu et al. investigated the positioning stage comprised of the ball-screw stage and 3-DOF piezoelectric stage [3]. In this system, the first stage ensures a large translation range and fast motion, while the second stage is responsible for the high-precision positioning. The stage uses piezoelectric actuators, as well as translation and rotation mechanisms to ensure proper operation. The system design, where high accuracy is achieved by using components based on flexure hinges and piezoelectric actuators, is efficient enough [4,5,6,7]. However, the main drawback of such systems is a complex structure and many components that must be controlled. Besides, piezoelectric stack actuators used in such systems are affected by hysteresis of piezoelectric material that must be evaluated by the control system of the device.

There are several multi-DOF ultrasonic actuators developed that are capable to position an object in two or three directions [7,8,9,10,11,12,13]. A 3-DOF Langevin type piezoelectric actuator was developed by Zhao et al. [9]. It is composed of a cylindrical stator and a spherical rotor. The actuator operates employing the superposition of bending and longitudinal vibration modes, when the excitation voltages with certain frequency, phase and amplitude are applied to the three groups of piezoelectric ceramic rings. A 3-DOF piezoelectric actuator generating rotation of the sphere about three axes has been developed by Vasiljev et al. [10]. The operation of the actuator is based on a shaking beam principle. The proposed actuator consists of a vibrating frame, four piezoceramic stacks and four overlays. Four driving tips are mounted on the top of the vibrating frame and are in contact with the sphere. Four electric signals with shifted phases by π/2 are used for excitation. Multiple degrees-of-freedom ultrasonic motor consisting of a bar-shaped stator and a spherical rotor has been reported [11]. Motor can generate 3-DOF rotation of the rotor around perpendicular axes using the bending and longitudinal vibration of the stator. A similar small-size cylinder-type multi-DOF actuator was developed by Gouda et al. [12]. The actuator consists of a small cylinder fixed on a substrate and a thin PZT ring that is glued to the substrate. The PZT electrodes are divided into four parts and are driven by shifted phase signals. A combination of the first longitudinal and the second bending mode is used to obtain elliptical vibrations on the top surface of the cylinder and to rotate a ball about three axes independently.

A multi-DOF cylindrical resonant-type piezoelectric actuator is presented and analyzed in this paper. This actuator has a triangular shape configuration of the electrodes and two active kinematic pairs. Compared to our previous research, an additional kinematic pair has been introduced and two additional degrees-of-freedom have been achieved, respectively [14,15]. This piezoelectric actuator can rotate the sphere about three axes and simultaneously move itself on the plane. The positioning of the sphere can be achieved through the direct impact of the sphere when the contacting point hits the sphere, or by moving it to the needed position on the plane. A combination of different voltages applied to electrodes creates an elliptical movement of the contact points. The mathematical model of contacting force control was developed for sphere trajectory planning. A numerical investigation of the actuator was performed, and a prototype actuator was manufactured and tested. The multi-DOF positioning system was realized using a single piezoelectric actuator that ensures higher accuracy. The proposed actuator can be applied for laser beam positioning in space, adaptive focusing of optical lenses in optoelectronics and laser systems, or optic beam stabilization.

## 2. Description of the Actuator and Modeling Results

**Figure 1:**
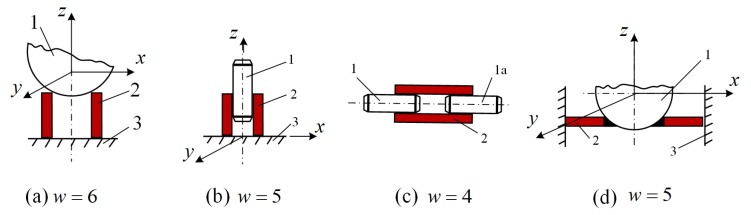
General schematics of the piezoelectric actuator with two active kinematic pairs.

Several principle design schemes of the piezoelectric actuator with two kinematic pairs are shown in Figure 1, where *w* is the total number of the degrees-of-freedom of moving links *1* and *1a*; *2* is the piezoelectric cylinder with special sectioning of electrodes, and *3* is a frame, on which additional displacements occur. Thus, the displacements of the moving link are quite evident: link *1* rotates around axes *x, y, z* and affects the translational motion in directions *x, y* and rotation around axis *z* (Figure 1a); link *1* rotates around axis *z*, affects translational motion in the direction of *z* axis and moves on the plane *x, y* (Figure 1b); links *1* and *1a* rotate around axis x and move along it (Figure 1c); link *1* rotates around axes *x, y, z* and affects translational motion in the direction of *z* axis and rotation around the same axis (Figure 1d).

**Figure 2:**
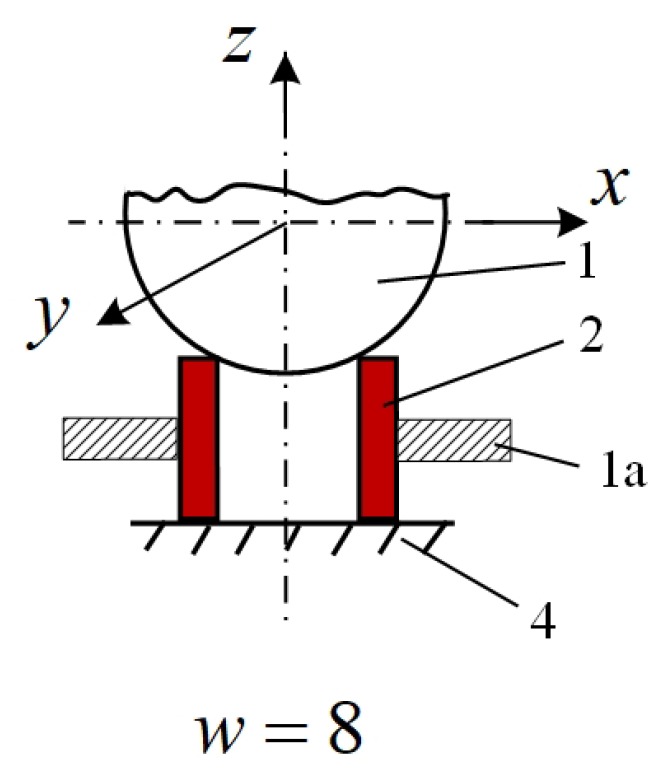
The schematic of the piezoelectric actuator with three active kinematic pairs.

The number of degrees-of-freedom could be increased: a schematic of the piezoelectric actuator with three active kinematic pairs (w=8) is shown in Figure 2. To obtain 8 degrees-of-freedom, it is necessary to solve the problem of electrode sectioning of the piezoelectric actuator with the aim to exclude the unwanted oscillations of some specific forms.

**Figure 3:**
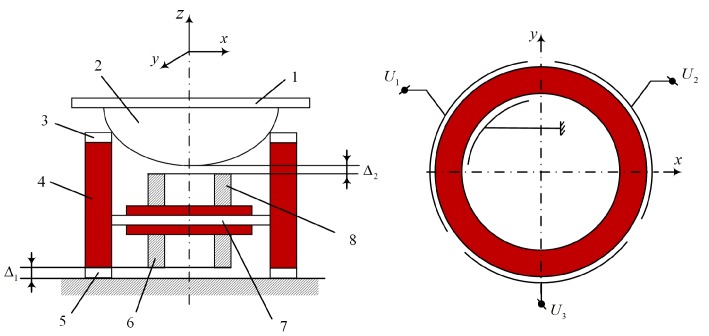
The laser deflector made on the basis of a single piezoelectric actuator with two active kinematic pairs and two brakes, activated with the help of a bimorph piezoelectric actuator, generating static bending displacements: *1*—mirror, *2*—spherical element, *3*—three contacts made of high friction material, *4*—piezoelectric cylinder with radial poling, *5*—three contacts made of high friction material, *6* and *8*— permanent magnets - brakes, fixed to bimorph actuator *7*, working in static displacement mode; $\Delta_{1}=0$, when the displacement of bimorph actuator *7* is negative (the brake is applied to the lower kinematic pair— displacements on the plane); $\Delta_{2}=0$, when the displacement of bimorph actuator *7* is positive (the brake is applied to the upper kinematic pair—angular displacements on the sphere).

Two types of motion (the sphere rotating on the cylinder and the sphere moving on the plane) can be controlled separately by:

- controlling the forces, acting in the contact zones of the sphere, piezoelectric cylinder and plane by introducing controllable brakes with the help of permanent magnets (Figure 3) or using layers of electro-rheological suspensions, controlled by high voltage
- connecting the same frequency voltage to specific electrodes, but with a different phase (180°) and amplitude, as in the main driving source
- using additional elements related to the position of the nodes of oscillation

**Figure 4:**
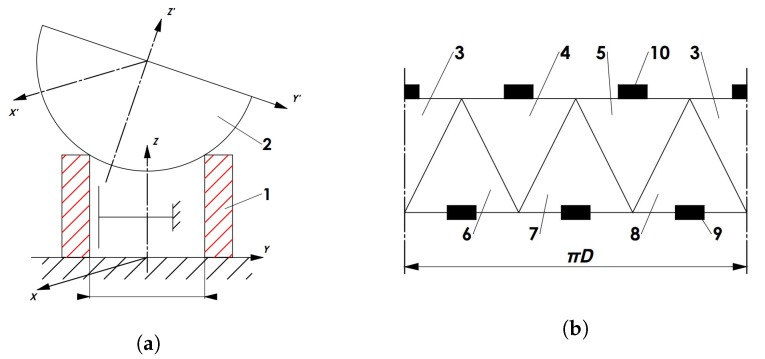
(**a**) Analyzed kinematic pair; (**b**) Proposed triangular electrode scheme.

We will further analyze the piezoelectric cylinder-type actuator with two kinematic pairs as shown in Figure 4a. A triangular electrode configuration is used to excite vibrations of the actuator (Figure 4b). The brakes of active kinematic pairs are not included. The piezoelectric cylinder has the following dimensions: 28 mm (inner diameter) × 33 mm (outer diameter) × 20 mm (height) and is made from PZT-8 material. A steel sphere is positioned on top of the cylinder (Figure 4a). The outer surface of the cylinder is covered with triangular electrodes as shown in Figure 4b, while the inner surface of the cylinder has a single electrode and is grounded.

Two electrodes on the outer surface are excited at the same time to achieve rotation of the rotor. Different voltages are applied to achieve the desired motion—larger voltage results in higher amplitude but not necessarily in the proper direction of the contact point. Therefore, improper voltages may result in elliptical trajectories of the contact points moving in opposite directions. It was chosen to apply a voltage of 100 V to the first electrode and 50 V to the second. Harmonic excitation signal was used. To determine proper resonance frequencies, the amplitudes of contact point vibration were investigated, when excitation frequency was changing in the range 0 Hz–150,000 Hz.

**Figure 5:**
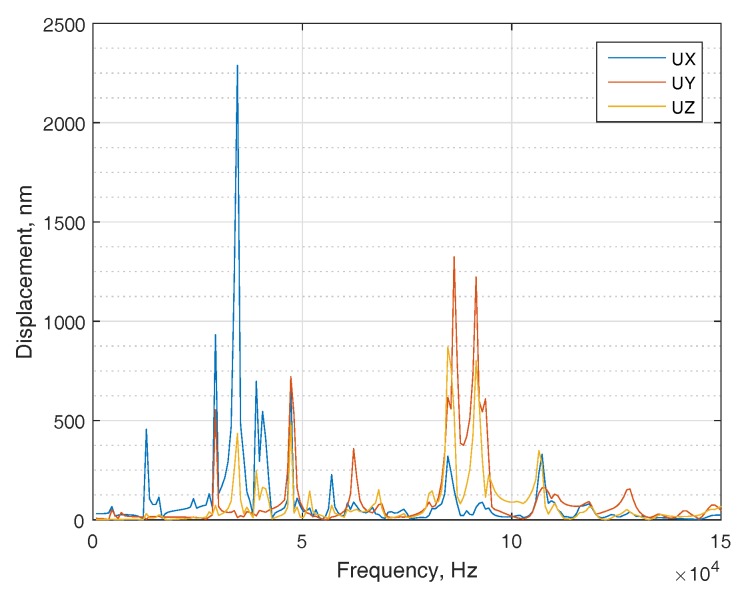
Harmonic analysis: The amplitudes of the contact point in the middle of electrode 4.

**Figure 6:**
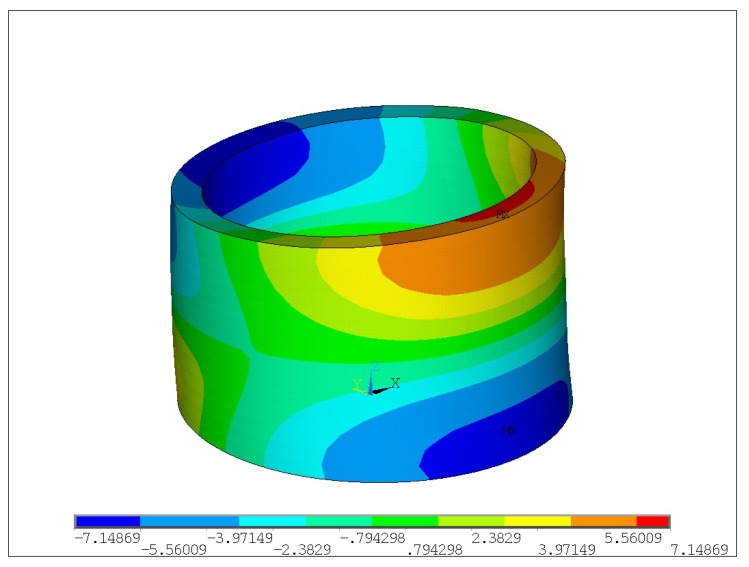
Modal analysis: The suitable eigenform at 88,371 Hz, deformations in Z direction.

The amplitudes of the contact point in the middle of electrode 4 (Figure 4b) are shown at Figure 5. The modal analysis shows that the required eigenform is at 88,371 Hz (Figure 6); therefore, a resonant frequency at 88,600 Hz will be further analyzed. This frequency resonates with the correct eigenform. The calculations and analysis were performed using ANSYS software.

**Figure 7:**
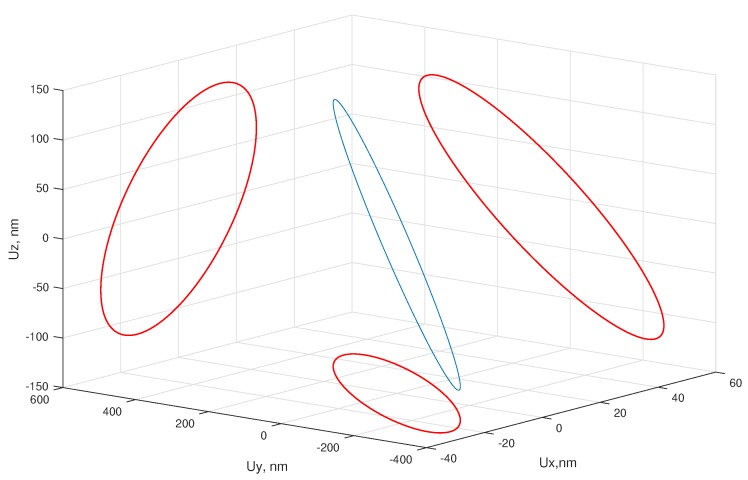
Trajectories of the contact point.

The proposed electrode configuration and the excitation of two electrodes with different voltages result in rotation of the sphere by two contact points. The contact point trajectory was calculated. The analysis shows that the contact point between electrodes 4 and 5 on *XZ* plane (Figure 1b) moves in ellipsis that has angle ϕ=deg88 (angle ϕ describes rotation of ellipse on the plane). The contact points that are positioned in the middle of electrodes 4 and 5 produce elliptical motion as well, but the angles are $\phi =74^{{}^{\circ}}$ and $\phi =82^{{}^{\circ}}$ respectively. The movement of the second contact point (Figure 4b—node 10) is shown in Figure 7. The direction and angles of the contact points can be effectively controlled by changing the applied voltage. When electrodes 6, 7 or 8 are excited, the biggest deformations occur in the bottom nodes. These are used to move the actuator on the plane. Exciting the top and bottom electrodes at the same time results in both rotations of the sphere and movement of the actuator.

## 3. Mathematical Model of the Contact Force Control

A mathematical model for sphere positioning in space through contacting force control was developed. One active kinematic pair of the actuator was used for sphere positioning on the plane. This kind of positioning is performed by moving the piezoelectric cylinder on the plane. Another active kinematic pair is used to rotate the sphere about three axes. All previously studied positioning systems had only one kinematic pair, i.e., the active cylinder was moved on the plane or the sphere was rotated about different axes. Combining two kinematic pairs and applying the brakes, as shown in Figure 3, we obtain one active cylinder with two kinematic pairs (Figure 4a). The configuration of electrodes should be as follows (Figure 4b):

- the sphere rotates on the cylinder and the cylinder does not move, when electrodes 3, 4, 5 are excited
- the cylinder moves on the plane and the sphere does not rotate, when electrodes 6, 7, 8 are excited

Different electrode switching and trajectory planning algorithms of the cylinder movement on the plane and sphere rotation are developed [16,17]. However, there is no research performed and trajectory planning algorithm developed, when two active kinematic pairs are used. Therefore, a new algorithm must be created. Based on the previous investigation of trajectory planning algorithms, it can be concluded that only a fine trajectory planning algorithm is suitable for this task [14,15]. The fine trajectory planning algorithm allows exciting two or more electrodes of the cylinder at the same time for obtaining a linear motion of the cylinder or rotation of the sphere. This method has the advantage of achieving the maximum available speed of movement, when certain contact forces are used. Therefore, the main task of the trajectory planning algorithm is to calculate the function of the contacting forces that can be obtained by exciting a particular electrode section of the piezoelectric cylinder.

**Figure 8:**
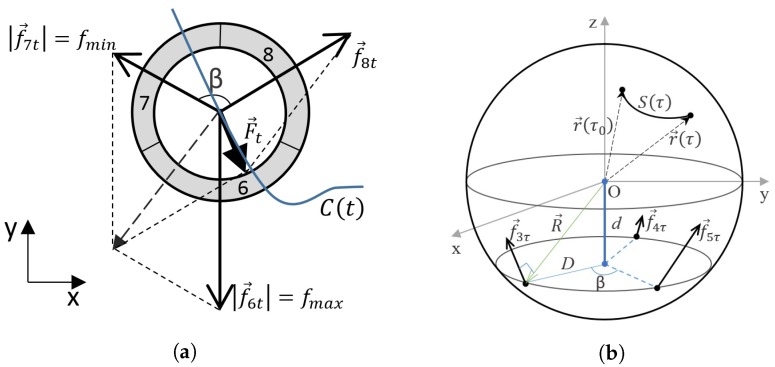
(**a**) Trajectory of the cylinder; (**b**) Sphere rotation trajectory.

The cylinder moves on the plane by the trajectory described as function C(t) (Figure 8a), the sphere rotates around its central axis by the trajectory described as function $S\left(\tau \right)=\vec{r}\left(\left|\vec{R}\right|,\theta \left(\tau \right),\phi \left(\tau \right)\right)$ (Figure 8b), where $\theta \left(\tau \right)\in \left[0,\pi \right]$ is an angle between positive *z* axis and $\vec{r}$; $\phi \left(\tau \right)\in \left[0,2\pi \right]$ is an angle between positive *x* axis and projection in xy plane of $\vec{r}$ (Figure 8); *t* and τ are parameters of the trajectories. Each kinematic pair is controlled at different value intervals:

(1) $$\begin{array}{c} \left|{\vec{f}}_{Jt}\right|\in \left[f_{min}^{t},f_{max}^{t}\right]\\ \left|{\vec{f}}_{J\tau }\right|\in \left[f_{min}^{\tau },f_{max}^{\tau }\right] \end{array}$$

The orientation of contact forces J=3,4,5 of the sphere does not change throughout the movement time:

(2) $$\vec{{f_{J}}^{{}^{\circ}}}=\left(\cos\left(\alpha_{J\tau }\right),\cos\left(\beta_{J\tau }\right),\cos\left(\gamma_{J\tau }\right)\right)$$

where $\alpha_{J}$,$\beta_{J}$,$\gamma_{J}$ is an angle between x,y,z axis and forces ${\vec{f}}_{J\tau }$. The rotation direction of the sphere is the same as the direction of the unit tangent vector at point τ of function S(τ). The unit tangent vector also shows the direction, in which the total force must be active. The unit tangent vector $\vec{T}\left(\tau \right)=\langle T_{x}|T_{y}|T_{z}\rangle$ is calculated by the formula [14]:

(3) $$\vec{T}\left(\tau \right)=\frac{{\vec{r}}^{\prime }\left(\tau \right)}{\left|{\vec{r}}^{\prime }\left(\tau \right)\right|}$$

The distance from the center of the sphere to the plane, where the contacts of the cylinder are located, are equal to $d=\sqrt{R^{2}-D^{2}}$, where *R* is the radius of the sphere; *D* is the radius of the cylinder and d<R. Then orientation of forces ${\vec{f}}_{3,4,5}$ of the sphere can be calculated by formula [15]:

(4) $$\left[{\vec{f}}_{J}\left(\alpha_{J},\beta_{J},\gamma_{J}\right)\right]=\cos^{-1}\left[\begin{array}{ccc} \frac{\sqrt{R^{2}-D^{2}}}{R} & 0 & \sqrt{D^{2}-R^{2}+1}\\ -\frac{\sqrt{R^{2}-D^{2}}}{2R} & \frac{\sqrt{3}}{2}\frac{\sqrt{R^{2}-D^{2}}}{R} & \sqrt{D^{2}-R^{2}+1}\\ -\frac{\sqrt{R^{2}-D^{2}}}{2R} & \cos\left(180^{{}^{\circ}}-\cos^{-1}\left(\frac{\sqrt{3}}{2}\frac{\sqrt{R^{2}-D^{2}}}{R}\right)\right) & \sqrt{D^{2}-R^{2}+1} \end{array}\right]$$

To choose an appropriate electrode segment for excitation, it is necessary to know the total force $\vec{F}$ of all segments:

(5) $$\begin{array}{c} {\vec{F}}^{C}\left(t\right)=\sum_{J=6}^{n}{\vec{f}}_{J}\left(t\right),\;\mathrm{in}\;\mathrm{case}\;J=6,7,8\;n=3;\\ {\vec{F}}^{C}\left(\tau \right)=\sum_{J=3}^{n}{\vec{f}}_{J}\left(\tau \right),\;\mathrm{in}\;\mathrm{case}\;J=3,4,5\;n=3; \end{array}$$

The electrode segment that is the nearest to the total force $\vec{F}$ vector must be selected for excitation and its numeric value must be equal to maximum $f_{max}$ (Figure 8a). To obtain precision of the motion, a force that is cardinally in the opposite orientation than the total force must be activated. Its value must be equal to the minimum value of the force $f_{min}$. The value of the third electrode is calculated after solving a system of equations [14]:

(6) $$\begin{array}{c} \left\{\begin{array}{c} \left|\vec{F}\right|\cos\mu =\sum_{J}^{n}\left|{\vec{f}}_{J}\right|\cos\phi_{J}\\ \left|\vec{F}\right|\sin\mu =\sum_{J}^{n}\left|{\vec{f}}_{J}\right|\sin\phi_{J} \end{array}\right.\\ \mathrm{where}\left|\vec{F}\right|=\tan^{-1}\frac{T_{y}}{T_{x}};\phi_{J}=\hat{\vec{F}{\vec{f}}_{J}}\;\mathrm{and}\;\left|\vec{f}\right|=\left\{\begin{array}{c} f_{min},\;\mathrm{if}\;\left|{\vec{f}}_{J}\right|=0,\\ f_{max},\;\mathrm{if}\;\phi_{J}=min\left(\left[\phi_{J}\right]\right)\\ \left|{\vec{f}}_{J}^{*}\right| \end{array}\right. \end{array}$$

**Figure 9:**
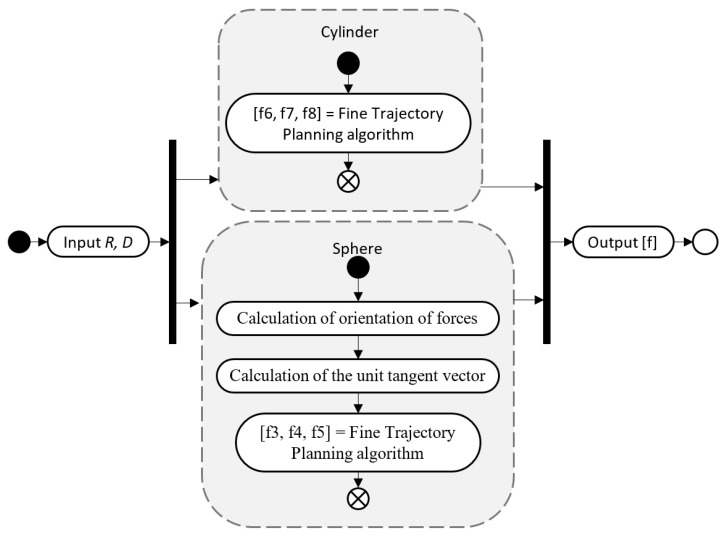
The integration system of the fine trajectory planning algorithm.

The integration scheme of the fine trajectory planning algorithm for one active element with two kinematic pairs, when parallel calculations are used, is presented in Figure 9.

The sphere of the radius *R* = 20 mm and the cylinder of the radius *D* = 16.5 mm were selected for the numerical experiment.

**Figure 10:**
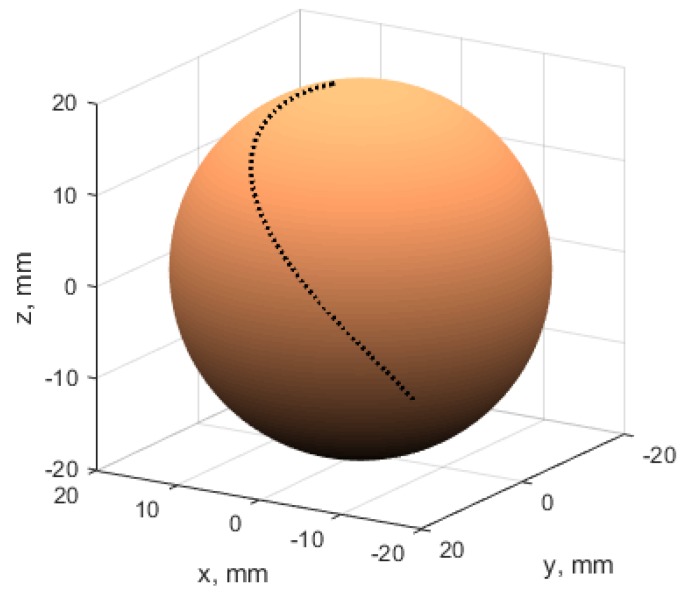
The sphere rotation trajectory (in dots).

The trajectory of the sphere rotation around different axes was described by formulas (Figure 10):

(7) $$S\left(\tau \right)=\left\{\begin{array}{c} \theta \left(\tau \right)=4\tau^{3}+10\\ \phi \left(\tau \right)=15\tau^{2}+2\tau \end{array}\right.,\;\;\;\mathrm{where}\;t\in \left[0,3\right]$$

(8) $$C\left(t\right)=\left\{\begin{array}{c} x(t)=t\cos(t)\\ y(t)=2t\sin(t) \end{array}\right.,\;\;\;\mathrm{where}\;t\in \left[0,3\right]$$

**Figure 11:**
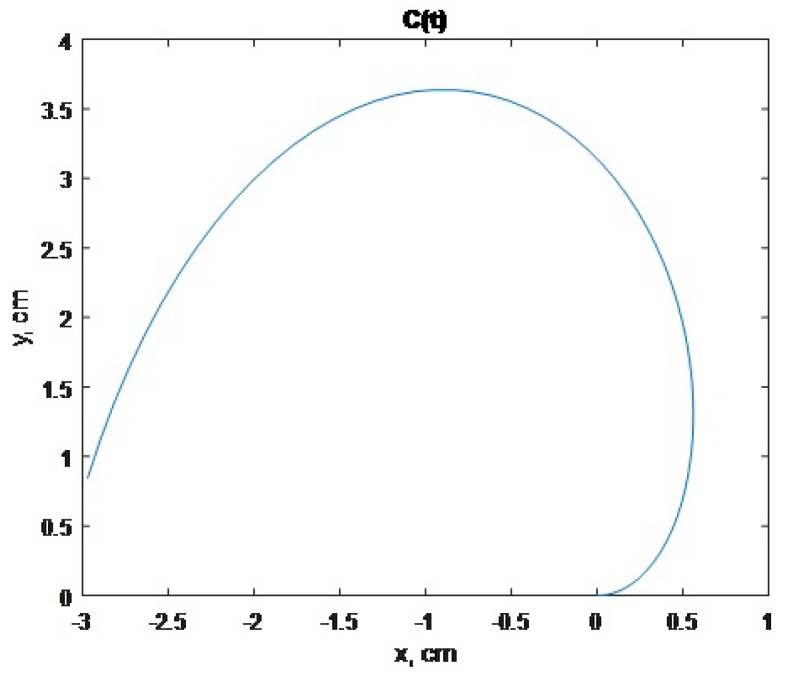
The cylinder motion trajectory.

**Figure 12:**
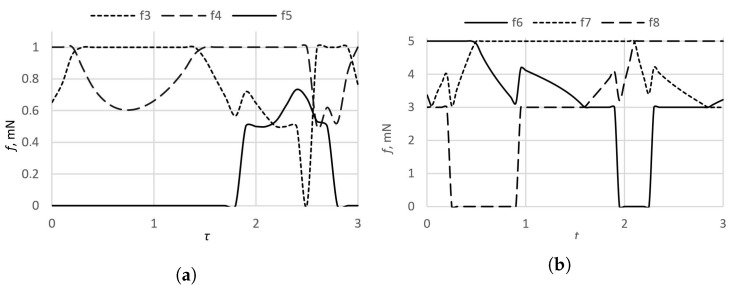
Forces of electrode segments: (**a**) the sphere; (**b**) the cylinder.

The trajetory of the cylinder is given in Figure 11. The forces of the sphere must be in the interval $\left|{\vec{f}}_{6,7,8}\right|\in \left[0.5,1\right]$ mN, and the forces of the cylinder in the interval $\left|{\vec{f}}_{3,4,5}\right|\in \left[3,5\right]$ mN. The results of the calculation of the cylinder forces and the sphere electrode segments are presented in Figure 12a,b.

The numerical experiment confirmed that the fine trajectory planning algorithm is successfully integrated and is suitable for both movements: the movement on the plane and sphere rotation on certain points of the contact. This means that kinematic pairs of the actuator can be managed separately.

## 4. Experimental Results

A radially poled piezoelectric cylinder with six triangle-shaped electrodes on the outer surface was produced based on the results of numerical simulations. The dimensions of the piezoelectric cylinder were 33 mm(outer diameter) × 28 mm (inner diameter) × 20 mm (height). The material was the hard type piezo ceramic of type PZT-8. Triangular electrodes on the outer surface of the piezoceramic cylinder were formed by etching using nitric acid.

**Figure 13:**
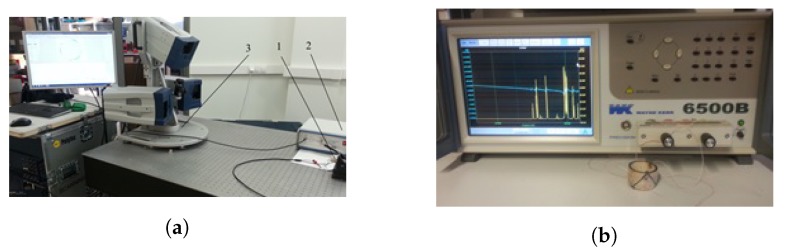
Experimental setup for investigating the modes of vibration (**a**) and impedance measurement of the piezoelectric cylinder (**b**): 1—piezoelectric cylinder; 2—linear amplifier; 3—3D scanning vibrometer; 4—impedance analyzer.

Measurements using a 3D scanning vibrometer and an impedance analyzer (Figure 13) are conducted to investigate the vibration modes of the piezoelectric actuator and to identify resonance frequencies. Equipment used for the experiment included a 3D scanning vibrometer PSV-500-3D-HV (Polytec GmbH, Springe, Germany), a linear amplifier P200 (FLC Electronics AB, Sweden) for the actuator driving and an impedance analyzer 6500B (Wayne Kerr Electronics Ltd., West Sussex, UK). The driving voltage of amplitude 5 V was a harmonic signal within the scanning frequency range of 10–150 kHz.

**Figure 14:**
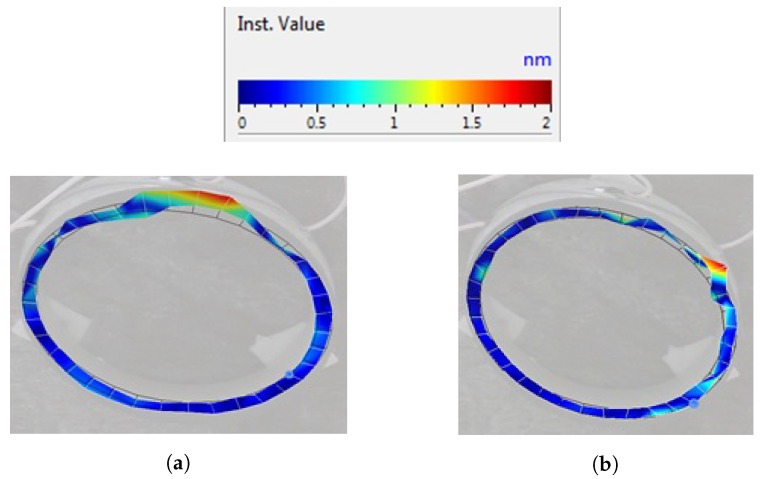
Deformation modes of the end side of piezoelectric cylinder, when an electric signal of the voltage amplitude of 5 V and a driving frequency of 93.6 kHz are applied into the triangle-shaped electrode: (**a**) the driving voltage is connected to the triangle-shaped electrode with the tip directed to the scanned surface; (**b**) the driving voltage is connected to the triangle-shaped electrode with the tip directed backwards to the scanned surface.

**Figure 15:**
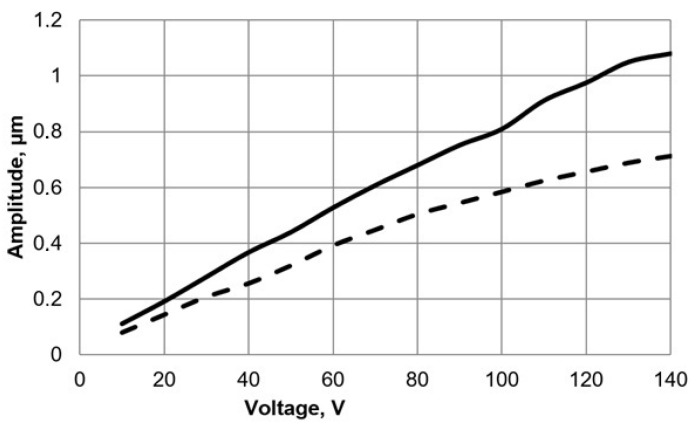
Amplitude vs applied voltage of the axial vibrations of contacting element at the operational frequency 93.6 kHz: dashed line—when the triangle-shaped electrode tip is directed to an active contacting element; solid line—when the triangle-shaped electrode tip is directed backwards to the active contacting element.

**Figure 16:**
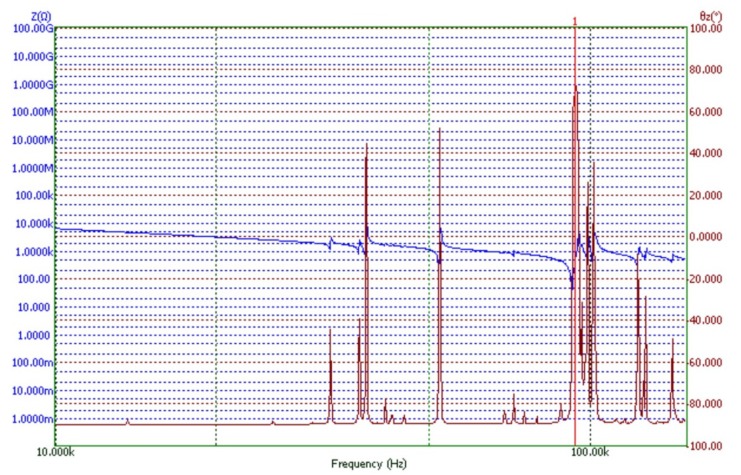
Impedance/phase diagrams of the piezoelectric cylinder. A resonant frequency for driving a transducer is 93.6 kHz.

**Figure 17:**
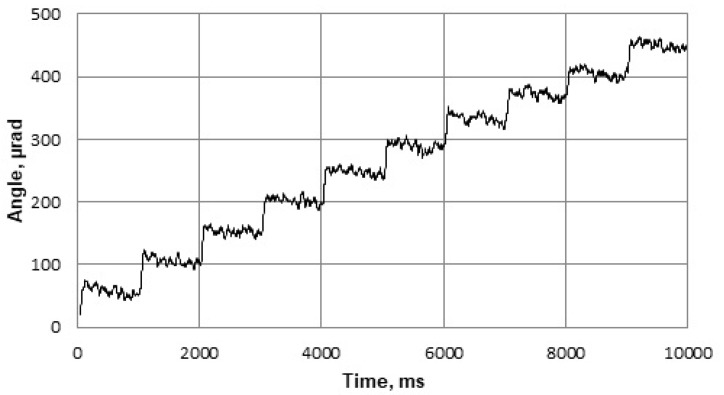
Measured stepped rotation of the sphere.

The vibrations of a cylinder-shaped transducer visualized with 3-D laser scanning vibrometer at a driving frequency of 93.6 kHz are shown in Figure 14. It can be noticed that the area of the deflections of the top surface is about 2 times larger, when driving voltage is connected to the triangle-shaped electrode with the tip directed backwards to the scanned surface. It confirms that the position of the 3 points of contact on the end surfaces of each side of the piezo cylinder should be by the middle of the side of the triangular electrode as shown in Figure 4b. Figure 15 shows dependence of the amplitude of the axial point of the contact point versus applied voltage. The dashed line shows the case when the triangle-shaped electrode with the tip directed to the active contacting element is excited; while solid line represents the case when the triangle-shaped electrode with the tip directed backwards to the active contacting element is excited. It can be noticed that almost linear dependence was obtained. Moreover, it can be seen that the vibration amplitude depends on electrode configuration, and larger amplitude value is obtained when the triangle-shaped electrode with the tip directed backwards to the active contacting element is excited. The results of the experiment show that three-dimensional resonant vibrations of contact points of the piezo cylinder are excited at a driving frequency of 93.6 kHz (Figure 16). These vibrations generate the lateral motion of the piezo cylinder placed on the horizontal plane, or the angular rotation of the sphere placed on 3 contact points of the piezo cylinder. The results of the rotation angle measurement about *x* axis of the sphere are shown in Figure 17. One electrode of the piezoelectric actuator was excited with the harmonic electric signal containing 10 periods. The excitation frequency was 93.6 kHz, while voltage amplitude was 40 V. Such electric signal was applied in steps every second. A minimal resolution of 50 μrad was obtained, whereas average rotation speed was 42.5 μrad/s. Compared to the results of FEM analysis, the difference of driving frequency differed only by 5.5% (88,361 Hz compared to 93,600 Hz). The direction of the lateral motion of the cylinder and the direction of the angular rotation of the sphere can be controlled by the algorithm presented in Section 3. A working prototype is shown in a Supplementary Video.

## 5. Conclusions

A new piezoelectric actuator with two active kinematic pairs was proposed and investigated. A novel electrode configuration scheme was used to excite vibrations of the actuator. Besides, a mathematical model of the contact force control and a trajectory planning algorithm were proposed and evaluated. Exciting different electrodes and controlling the contact force in kinematic pairs with brakes can create rotation of the sphere or movement on the plane. The trajectory planning method was validated by the numerical analysis. A prototype actuator was made, and mechanical and electrical characteristics were measured. The results of the numerical simulation and experimental measurements were in a good agreement.

## Supplementary Materials

The following are available online at http://www.mdpi.com/2072-666X/9/11/597/s1. Video S1: A motion of the sphere when driving voltage is applied to the actuator.

## References

[1] Cai K., Tian Y., Wang F., Zhang D., Liu X., Shirinzadeh B. (2017). Design and control of a 6-degree-of-freedom precision positioning system. Robot. Comput. Integr. Manuf..

[2] Zhu W., Yang F., Rui X. (2018). Robust independent modal space control of a coupled nano-positioning piezo-stage. Mech. Syst. Signal Process..

[3] Liu C.H., Jywe W.Y., Jeng Y.R., Hsu T.H., Li Y.T. (2010). Design and control of a long-traveling nano-positioning stage. Precis. Eng..

[4] Guo Z., Tian Y., Liu C., Wang F., Liu X., Shirinzadeh B., Zhang D. (2015). Design and control methodology of a 3-DOF flexure-based mechanism for micro/nano-positioning. Robot. Comput.-Integr. Manuf..

[5] Chen J., Xu M.L., Feng B., Tian Q.G. (2014). A novel positioning stage using piezoelectric actuator for antenna pointing. Recent Advances in Structural Integrity Analysis, Proceedings of the International Congress APCFS/SIF 2014.

[6] Liu P., Yan P., Zhang Z., Leng T. (2015). Modeling and control of a novel X-Y parallel piezoelectric-actuator driven nanopositioner. ISA Trans..

[7] Liaw H.C., Shirinzadeh B. (2010). Constrained motion tracking control of piezo-actuated flexure-based four-bar mechanisms for micro/nano manipulation. IEEE Trans. Autom. Sci. Eng..

[8] Gozen B.A., Ozdoganlar B. (2012). A method for open-loop control of dynamic motions of piezo-stack actuators. Sens. Actuators A Phys..

[9] Zhao C., Li Z., Huang W. (2005). Optimal design of the stator of a three-DOF ultrasonic motor. Sens. Actuator A Phys..

[10] Vasiljev P., Mazeika D., Kulvietis G., Vaiciuliene S. (2006). Piezoelectric Actuator Generating 3D-Rotations of the Sphere. Solid State Phenom..

[11] Aoyagi M., Beeby S.P., White N.M. (2002). A novel multi-degree-of-freedom thick-film ultrasonic motor. IEEE Trans. Ultrason. Ferroelectr. Freq. Control.

[12] Gouda Y., Nakamura K., Ueha S. (2006). A miniaturization of the multi-degree-of-freedom ultrasonic actuator using a small cylinder fixed on a substrate. Ultrasonics.

[13] Zhao C. (2011). Ultrasonic Motors: Technologies and Applications.

[14] Bansevicius R.P., Drukteiniene A., Kulvietis G., Macerauskas E., Janutenaite J., Mazeika D. (2016). Fine trajectory planning method for mobile piezorobots. J. Vibroeng..

[15] Bansevicius R.P., Genadijus K., Macerauskas E., Janutenaite J., Drukteiniene A., Mazeika D. Trajectory planning for stabilization system of nanosatellite. Proceedings of the Mechanika, 21th International Scientific Conference.

[16] Janutnaite-Bogdaniene J., Macerauskas E., Drukteiniene A., Kulvietis G., Bansevicius R.P. (2017). Cylindrical piezorobot’s trajectory planning and control. J. Vibroeng..

[17] Bansevicius R.P., Mazeika D., Kulvietis G., Tumasoniene I., Drukteiniene A., Jurenas V., Bakanauskas V. (2018). Investigation of sphere trajectories of a rotational type piezoelectric deflector. Mech. Syst. Signal Process..

